# Efficacy and Safety of Remimazolam in Endoscopic Sedation—A Systematic Review and Meta-Analysis

**DOI:** 10.3389/fmed.2021.655042

**Published:** 2021-07-26

**Authors:** Xianlin Zhu, Hongbai Wang, Su Yuan, Yinan Li, Yuan Jia, Zhe Zhang, Fuxia Yan, Zaiping Wang

**Affiliations:** ^1^Department of Anesthesiology, The Central Hospital of Enshi Tujia and Miao Autonomous Prefecture, Enshi City, China; ^2^Department of Anesthesiology, Fuwai Hospital, Chinese Academy of Medical Sciences and Peking Union Medical College, Beijing, China

**Keywords:** remimazolam, endoscopy, procedural sedation, adult, meta-analysis

## Abstract

**Background:** The aim of this systematic review and meta-analysis was to investigate the efficacy and safety of remimazolam in clinical endoscopic procedure sedation.

**Methods:** The authors searched the databases of PubMed, Embase, and Cochrane Library for studies published until January 2, 2021, that reported remimazolam sedation for endoscopic procedures. The sedative efficiency and the incidence of adverse events were assessed as outcomes. Cochrane Review Manager Software 5.3 was used to perform the statistical analyses.

**Results:** Seven relevant studies involving a total of 1,996 patients were identified. We conducted a meta-analysis of the different controls used in the studies, that is, the placebo, midazolam, and propofol. The results demonstrated that remimazolam had a strong sedative effect, and its sedative efficiency was significantly higher than that of placebo [OR = 0.01, 95% CI: (0.00, 0.10), *I*^2^ = 30%, *p* <0.00001]. The sedative efficiency of remimazolam was significantly higher than that of midazolam [OR = 0.12, 95% CI: (0.08, 0.21), *I*^2^ = 0%, *p* < 0.00001] but lesser than that of propofol [OR = 12.22, 95% CI: (1.58, 94.47), *I*^2^ = 0%, *p* = 0.02]. Regarding the adverse events, remimazolam is associated with a lower incidence of hypotension than placebo and midazolam. Similarly, remimazolam was associated with a lower incidence of hypotension and hypoxemia than propofol.

**Conclusions:** Remimazolam is a safe and effective sedative for patients undergoing endoscopic procedures. The sedative efficiency of remimazolam was significantly higher than that of midazolam but slightly lower than that of propofol. However, the respiration and circulation inhibitory effects of remimazolam were weaker than those of midazolam and propofol.

## Introduction

Endoscopy, including gastrointestinal endoscopy, bronchoscopy, and other types of endoscopy, is the most convenient, safe, and effective method for detecting gastrointestinal or bronchial hemorrhage, tumors, and precancerous lesions. It has been widely used in clinical practice (1). Millions of patients receive endoscopy each year because of digestive tract and other disorders globally. However, endoscopy is an invasive procedure, and patients may have several forms of discomfort such as nervousness, fear, cough, gastrointestinal spasm, and severe complications such as arrhythmia and cerebrovascular accidents (2, 3).

Compared with traditional endoscopy, the use of sedatives and analgesics during endoscopy can eliminate fear and relieve pain in patients, as well as reduce the difficulty of the endoscopic procedure and shorten the duration of the procedure (4, 5). At present, the sedative drugs used in clinical endoscopy are mainly midazolam and propofol. Midazolam has a long duration of action and slow recovery from anesthesia (6, 7). In addition to the injection site pain, propofol also has strong respiratory and circulatory inhibitory effects, thus increasing the incidence of accidental risks such as hypoxemia, hypotension, and cardiac arrest (8, 9).

Remimazolam, an analog of midazolam, is a benzodiazepine and a new ultra-short-acting sedative (10, 11). Compared with midazolam, remimazolam has the advantages of rapid onset, rapid recovery, and a higher safety profile (12, 13). Previous studies have found that remimazolam has the same success rate of sedation as propofol but is associated with a lower incidence of hypotension and hypoxemia, and faster awakening time when used for endoscopic sedation (14). However, it is a new drug, and its efficacy and safety for endoscopic sedation have not been established. Therefore, we collected previously published relevant data to conduct a systematic review and meta-analysis on the efficacy and safety of remimazolam sedation for endoscopy.

## Materials and Methods

This systematic review and meta-analysis adhered to the Preferred Reporting Items for Systematic Reviews and Meta-Analyses (PRISMA) (Supplementary Table 1) (15).

### Search Strategy

Xianlin Zhu and Hongbai Wang were responsible for document retrieval. We searched the databases of Cochrane Library, Embase, and PubMed using the PICOS (Population, Intervention, Comparison, Outcome, Study design) method. The deadline for our search was January 2, 2021. The search terms included “Remimazolam” OR “CNS 7056” AND “Endoscopy” OR “Bronchoscopy” OR “Colonoscopy” OR “Gastroscopy,” and the search scope was “title and abstract.” We sought to evaluate all studies on the efficacy and safety of remimazolam for endoscopy, and we did not restrict the search to control drugs and specific study designs. Articles published in various languages were included. A manual search of the reference lists of reviews and research papers was conducted to exclude missing RCTs.

### Study Selection

Hongbai Wang and Yuan Jia screened the titles and abstracts, while Xianlin Zhu and Su Yuan screened the full texts. The inclusion criteria included the following: (1) participants undergoing endoscopic procedures, including gastroscopy, colonoscopy, gastrointestinal endoscopy, and bronchoscopy; and (2) sedation with remimazolam and placebo or other positive control agents. The exclusion criteria included (1) participants undergoing endoscopic procedures with anesthetics that could not be established; (2) duplicate articles; (3) review or meta-analysis; (4) basic research; (5) articles published as an abstract, editorial, case report, letter, note, conference article, method, or protocol; and (6) articles presented in a non-English language.

### Outcome Measures

The primary outcome was the sedative efficiency of remimazolam in endoscopy, and the secondary outcomes were the incidence of adverse events, including hypotension, hypoxia, bradycardia, nausea, vomiting, and pain of the injection site.

### Data Extraction

Yinan Li and Zhe Zhang were responsible for extracting the following information: (1) author; (2) publication year; (3) the number of participants in each study; (4) country of publication; (5) age range of all the participants; (6) gender composition; (7) the procedures that participants underwent; (8) the specific interventions that participants received, including the drug name, dose, and the medication regimen; (9) the methods and criteria for sedative efficacy assessment; and (10) the number of patients in the remimazolam and control group. Yinan Li extracted those data, and Zhe Zhang checked the extracted data.

### Quality Assessment of Included Studies

Fuxia Yan and Zaiping Wang independently assessed the methodological quality of the included studies. Since the included studies were all RCTs, and there were no retrospective or prospective observational studies in this systematic review and meta-analysis, the risk of bias was assessed using the Cochrane Collaboration Risk of Bias Assessment tool. They included the following seven items: random sequence generation (selection bias), allocation concealment (selection bias), blinding of participants and personnel (performance bias), blinding of outcome assessment (detection bias), incomplete outcome data (attrition bias), selective reporting (reporting bias), and others (bias due to vested financial interest and academic bias). If the study had one or more items associated with a high or unclear risk of bias, it was classified as high risk (16). If the two authors disagreed on their assessments, the corresponding author resolved any discrepancies to eliminate bias.

### Data Analysis

The Cochrane Review Manager Software (RevMan 5.3, Cochrane Collaboration, Oxford, UK) and Stata version 12.0 (Stata Corp, College Station, TX, USA) were used for the statistical analyses. We used the values of *I*^2^ and the Mantel–Haenszel chi-square test (*p*-value for heterogeneity) to assess inter-study heterogeneity. *I*^2^ <40%, 40 ≤ *I*^2^ <60%, and *I*^2^ ≥ 60% indicated low, moderate, and high heterogeneity, respectively (17). If significant heterogeneity was detected (*I*^2^ ≥ 50%), a leave-one-out sensitivity analysis was performed to assess the single comparison-driven inference. The meta-analysis was performed with a random-effects model when there was significant heterogeneity (*I*^2^ ≥ 50% or a *p*-value for heterogeneity < 0.1); otherwise, a fixed-effect model was used (*I*^2^ <50% or a *p*-value for heterogeneity ≥ 0.1) (18). The dichotomous outcome was reported as the odds ratios (OR) with a 95% confidence interval (CI). The statistical tests were two-sided, and a *p*-value for the overall effect of < 0.05 denoted significant differences.

## Results

### Study Selection

**Figure 1:**
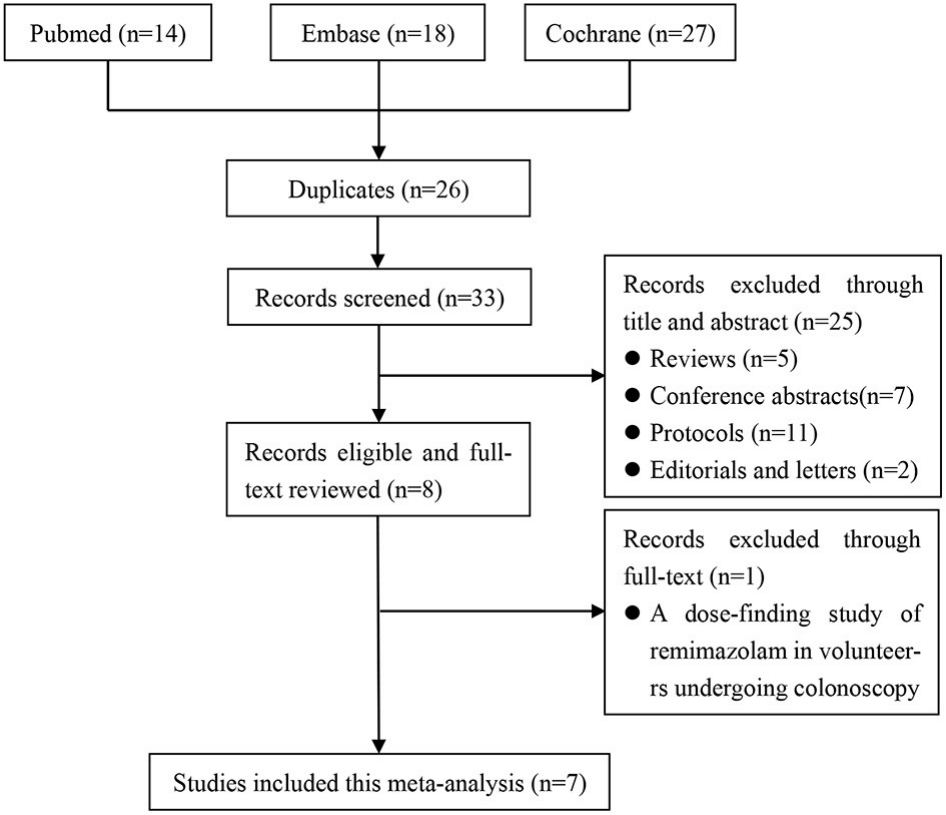
The screening process of the eligible literatures.

The literature search identified 59 potentially eligible articles: 14 from PubMed, 18 from Embase, and 27 from Cochrane Library. We removed 26 duplicate articles and excluded 25 articles at the title-and-abstract review stage according to the exclusion criteria. In addition, we excluded one trial at the full-text review stage; it was a dose-finding study of remimazolam involving volunteers undergoing colonoscopy, and it assessed the antagonistic effect of flumazenil in reversing remimazolam sedation (19). As illustrated in the PRISMA flow diagram, the final analysis included seven studies involving a total of 1996 patients (12–14, 20–23) (Figure 1).

### Studies and Participants' Characteristics

Seven studies involving a total of 1,996 patients were included, and all of them were RCTs (published April 2005–Jan 2021); four involved 1,079 patients undergoing colonoscopy (13, 21–23), two involved 478 patients undergoing upper gastrointestinal endoscopy (14, 20), and one involved 439 patients undergoing bronchoscopy (12). The age of the patients ranged from 18 to 95 years, and male patients accounted for 45.38% (Table 1). Seven studies adopted the same or similar criteria to assess sedative efficiency. We allocated the patients in each study to two groups according to the type of sedative drugs used for endoscopy: the remimazolam group and control groups (including placebo, midazolam, and propofol). The proportion of patients with successful sedation was 1,071/1,208 in the remimazolam group and 481/788 in the control group (placebo 4/139, midazolam 88/270, and propofol 379/379, respectively) (Table 2). In addition, the incidence of adverse events, especially hypotension and hypoxia, were widely recorded (Table 3).

**Table 1:** The basic characteristics of included studies.

**References**	**Study design**	**No. of patients**	**Country/centers**	**Procedures**	**Age (years)**	**Gender (M/F)**	**Criterion of sedation**	**Remimazolam**	**Control**
Borkett et al. (20)	RCT	100	United States/multicenter	Upper gastrointestinal endoscopy	18–65	46/54	Initiated sedation: MOAA/S ≤ 3; Maintained sedation: MOAA/S ≤ 4	Single dose: 0.10 mg/kg 0.15 mg/kg 0.20 mg/kg	Midazolam: (Single dose 0.075 mg/kg)
Chen et al. (21)	RCT	384	China/multicenter	Colonoscopy	18–65	161/223	Initiated sedation: MOAA/S ≤ 3; Maintained sedation: MOAA/S ≤ 4	Initial dose: 5.0 mg; Top-up dose: 2.5 mg per time	Propofol: (Initial dose: 1.5 mg/kg; top-up dose: 0.5 mg/kg per time)
Chen^*^ et al. (14)	RCT	378	China/multicenter	Upper gastrointestinal endoscopy	18–60	148/230	Initiated sedation: MOAA/S ≤ 3; Maintained sedation: MOAA/S ≤ 4	Initial dose: 5.0 mg; Top-up dose: 2.5 mg per time.	Propofol: (Initial dose:1.5 mg/kg; top-up dose:0.5 mg/kg per time)
Pambianco et al. (24)	RCT	160	United States/multicenter	Colonoscopy	18–70	72/88	Initiated sedation: MOAA/S ≤ 3; Maintained sedation: MOAA/S ≤ 4	Initial and top-up dose: 8.0/3.0 mg 7.0/2.0 mg 5.0/3.0 mg	Midazolam: (Initial and top-up dose: 2.5/1.0 mg)
Pastis et al. (12)	RCT	439	United States/multicenter	Bronchoscopy	22–95	206/233	Initiated sedation: MOAA/S ≤ 3; Maintained sedation: MOAA/S ≤ 4	Initial dose: 5.0 mg; Top-up dose: 2.5 mg per time	Placebo; Midazolam: (Initial dose 1.75 mg <60 years or 1.0 mg > 60 years; top-up dose: 1.0 mg <60 years or 0.5 mg > 60 years)
Rex et al. (13)	RCT	458	United States/multicenter	Colonoscopy	19–92	226/232	Initiated sedation: MOAA/S ≤ 3; Maintained sedation: MOAA/S ≤ 4	Initial dose: 5.0 mg; Top-up dose: 2.5 mg per time	Placebo; Midazolam: (Initial dose 1.75 mg <60 years or 1.0 mg > 60 years; top-up dose: 1.0 mg <60 years or 0.5 mg > 60 years)
Rex et al. (13)	RCT	77	United States/multicenter	Colonoscopy	42–84	43/34	Initiated sedation: MOAA/S ≤ 3; Maintained sedation: MOAA/S ≤ 4	Initial dose: 2.5–5.0 mg; Top-up dose: 1.25–2.5 mg	Placebo; Midazolam: (Initial dose 1.0 mg; top-up dose: 0.5 mg)

*MOAA/S, Modified Observer's Assessment of Alertness/Sedation. The evaluation criteria: responds readily to name spoken in normal tone (alert, 5 score); lethargic response to name spoken in normal tone (4 score); responds only after name is called loudly or repeatedly (3 score); responds only after mild prodding or shaking (2 score); does not respond to mild prodding or shaking (1 score); does not respond to noxious stimulation (0 score)*.

* *indicates different articles published by the same author in the same year*.

**Table 2:** The number of patients with successful sedation and assessment methods of successful sedation in endoscopy.

**References**	**Study design**	**No. of patients in each group**		**No. of successful sedation**		**Assessment methods of successful sedation**
		**Remimazolam**	**Control**	**Remimazolam**	**Control**	
Borkett et al. (20)	RCT	0.10 mg/kg: 25 0.15 mg/kg: 25 0.20 mg/kg: 25	Midazolam: 25	0.10 mg/kg: 8 0.15 mg/kg: 14 0.20 mg/kg: 16	Midazolam: 14	(1), (2), (3), (5)
Chen et al. (21)	RCT	194	Propofol:190	188	Propofol: 190	(2), (3), (4)
Chen^*^ et al. (14)	RCT	189	Propofol: 189	184	Propofol: 189	(2), (3), (4)
Pambianco et al. (24)	RCT	8.0/3.0 mg: 40 7.0/2.0 mg: 40 5.0/3.0 mg: 40	Midazolam: 40	8.0/3.0 mg: 37 7.0/2.0 mg: 38 5.0/3.0 mg: 39	Midazolam: 30	(1), (2), (3), (5)
Pastis et al. (12)	RCT	303	Placebo: 63 Midazolam: 73	250	Placebo: 3 Midazolam: 24	(2), (3), (4)
Rex et al. (13)	RCT	296	Placebo: 60 Midazolam: 102	270	Placebo: 1 Midazolam: 26	(2), (3), (4)
Rex et al. (13)	RCT	31	Placebo: 16 Midazolam: 30	27	Placebo: 0 Midazolam: 4	(2), (3), (4)

*The successful sedation was defined as follows: (1) MOAA/S ≤ 4 on three consecutive measurements taken every minute; (2) completion of the whole endoscopy procedure; (3) no requirement for an alternative and/or rescue sedative; (4) administered up to a maximum of five supplemental doses within 15 min after the initial dose; (5) no manual or mechanical ventilation*.

* *indicates different articles published by the same author in the same year*.

**Table 3:** The number of patients with adverse events during endoscopy.

**References**	**Patients in each group (*****n*****)**		**Hypotension (*****n*****)**		**Hypoxia (*****n*****)**		**Bradycardia (*****n*****)**	
	**Remimazolam**	**Control**	**Remimazolam**	**Control**	**Remimazolam**	**Control**	**Remimazolam**	**Control**
Borkett et al. (20)	0.10 mg/kg: 25 0.15 mg/kg: 25 0.20 mg/kg: 25	Midazolam: 25	0.10 mg/kg: 0 0.15 mg/kg: 0 0.20 mg/kg: 0	Midazolam: 1	0.10 mg/kg: 4 0.15 mg/kg: 5 0.20 mg/kg: 6	Midazolam:5	NA	NA
Chen et al. (21)	194	Propofol: 190	46	Propofol: 97	6	Propofol: 32	2	Propofol: 7
Chen^*^ et al. (14)	189	Propofol: 189	24	Propofol: 81	2	Propofol: 13	NA	NA
Pambianco et al. (24)	8.0/3.0 mg: 40 7.0/2.0 mg: 40 5.0/3.0 mg: 40	Midazolam: 40	8.0/3.0 mg: 1 7.0/2.0 mg: 1 5.0/3.0 mg: 0	Midazolam: 0	8.0/3.0 mg: 1 7.0/2.0 mg: 2 5.0/3.0 mg: 0	Midazolam: 1	NA	NA
Pastis et al. (12)	303	Placebo: 59 Midazolam: 69	127	Placebo: 37 Midazolam: 34	66	Placebo: 12 Midazolam: 13	13	Placebo: 4 Midazolam: 3
Rex et al. (13)	296	Placebo: 60 Midazolam: 102	115	Placebo: 25 Midazolam: 63	3	Placebo: 2 Midazolam: 1	33	Placebo: 7 Midazolam: 16
Rex et al. (13)	31	Placebo: 16 Midazolam: 30	18	Placebo: 11 Midazolam: 17	6	Placebo: 2 Midazolam: 8	1	Placebo: 1 Midazolam: 4
**Study**	**Patients in each group (*****n*****)**		**Nausea (*****n*****)**		**Vomiting (*****n*****)**		**Pain of injection site (*****n*****)**	
	**Remimazolam**	**Control**	**Remimazolam**	**Control**	**Remimazolam**	**Control**	**Remimazolam**	**Control**
Borkett et al. (20)	0.10 mg/kg: 25 0.15 mg/kg: 25 0.20 mg/kg: 25	Midazolam: 25	NA	NA	NA	NA	NA	NA
Chen et al. (21)	194	Propofol: 190	5	Propofol: 1	1	Propofol: 0	1	Propofol: 19
Chen^*^ et al. (14)	189	Propofol: 189	NA	NA	NA	NA	0	Propofol: 31
Pambianco et al. (24)	8.0/3.0 mg: 40 7.0/2.0 mg: 40 5.0/3.0 mg: 40	Midazolam: 40	NA	NA	NA	NA	NA	NA
Pastis et al. (12)	303	Placebo: 59 Midazolam: 69	12	Placebo: 2 Midazolam: 2	6	Placebo: 1 Midazolam:2	2	Placebo: 0 Midazolam: 0
Rex et al. (13)	296	Placebo: 60 Midazolam: 102	5	Placebo: 4 Midazolam: 2	3	Placebo: 2 Midazolam: 0	NA	NA
Rex et al. (13)	31	Placebo: 16 Midazolam: 30	NA	NA	NA	NA	NA	NA

* *indicates different articles published by the same author in the same year*.

### Risk of Bias Assessment

**Figure 2:**
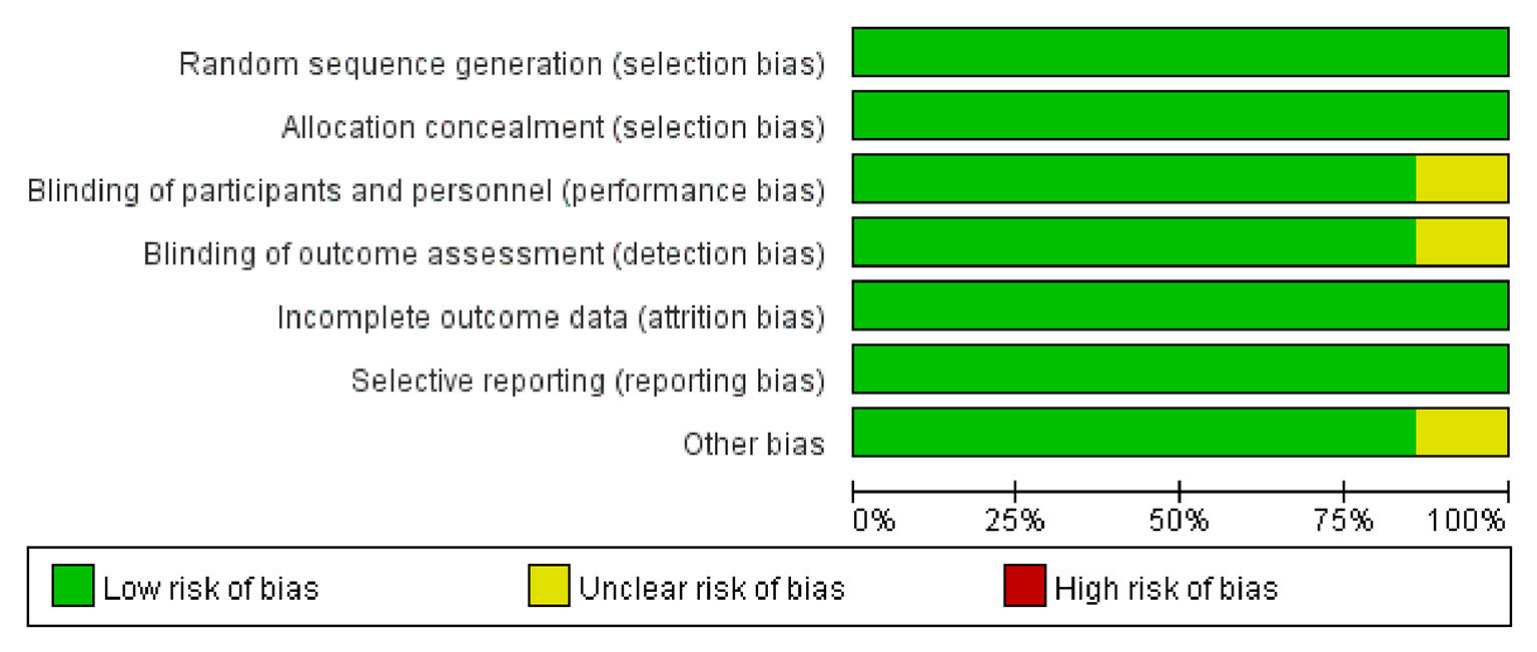
The risk of bias graph of included studies.

**Figure 3:**
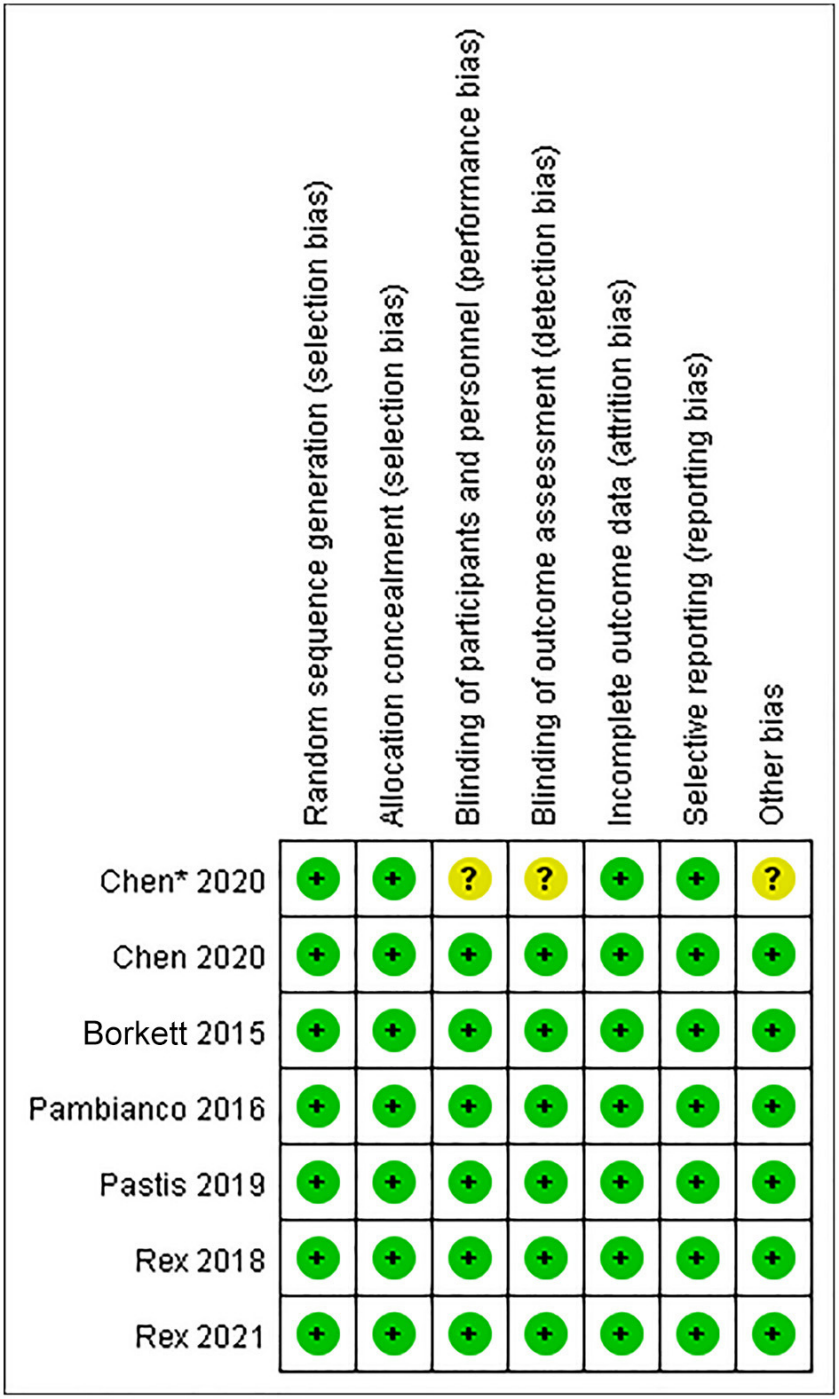
The risk of bias summary of included studies.

The Cochrane Collaboration Risk of Bias Assessment tool was used to assess the risk of bias for the RCTs. The included seven studies demonstrated a low risk of bias, as they assessed the random sequence generation (seven studies, 100%), allocation concealment (seven studies, 100%), blinding of participants and personnel (six studies, 85.7%), blinding of outcome assessment (six studies, 85.7%), incomplete outcome data (seven studies, 100%), selective reporting (seven studies, 100%), and others (six studies, 85.7%). Among these studies, six studies were found to be of high quality (Figures 2, 3).

### The Sedative Efficiency

**Figure 4:**
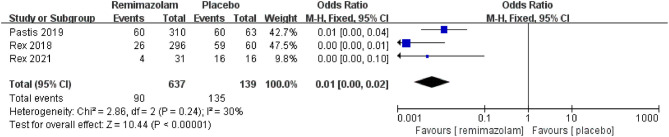
The comparison of sedative efficacy between remimazolam and placebo.

**Figure 5:**
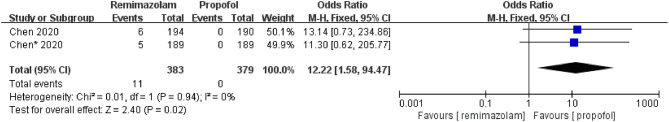
The comparison of sedative efficacy between remimazolam and propofol.

**Figure 6:**
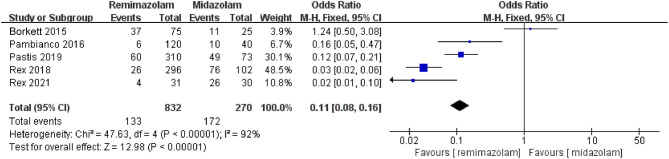
The pooled results of sedative efficacy between remimazolam and midazolam before the sensitivity analysis.

**Figure 7:**
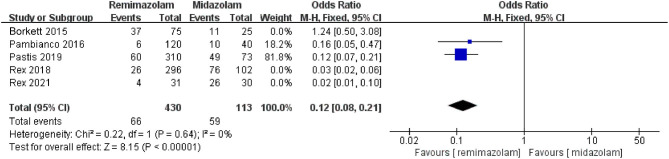
The pooled results of sedative efficacy between remimazolam and midazolam after the sensitivity analysis.

Three studies involving 776 patients have compared the sedative efficacies of remimazolam and placebo (remimazolam group, *n* = 637; placebo group, *n* = 139). The pooled results demonstrated significant differences between the two groups, and the sedative efficacy was higher in the remimazolam group [OR = 0.01, 95% CI: (0.00, 0.10), *I*^2^ = 30%, *p* < 0.00001] (Figure 4). Two studies involving 762 patients have compared the sedative efficacies of remimazolam and propofol (remimazolam group, *n* = 383; propofol group, *n* = 379). The pooled results demonstrated significant differences between two groups, and sedative efficacy was higher in the propofol group [OR = 12.22, 95% CI: (1.58, 94.47), *I*^2^ = 0%, *p* = 0.02] (Figure 5). Five studies involving 1,102 patients have compared the sedative efficacies of remimazolam and midazolam (remimazolam group, *n* = 832; midazolam group, *n* = 270). The pooled results demonstrated significant differences between two groups [OR = 0.11, 95% CI: (0.08, 0.16), *I*^2^ = 92%, *p* < 0.00001] (Figure 6). Due to the noted significant heterogeneity between the included studies (*I*^2^ = 92%), a leave-one-out analysis was performed. When the three studies (13, 20), were excluded from the analysis, there was still a significant difference between the two groups, and sedative efficacy favored the remimazolam group [OR = 0.12, 95% CI: (0.08, 0.21), *I*^2^ = 0%, *p* < 0.00001] (Figure 7).

### The Incidence of Adverse Events

The pooled results demonstrated significant differences between the remimazolam and placebo groups related to the incidence of hypotension; the remimazolam group showed a better outcome [OR = 0.62, 95% CI (0.42, 0.91), *I*^2^ = 36%, *p* = 0.01]. There was no difference between the two groups based on the incidence of hypoxia, bradycardia, nausea, vomiting, and pain at the injection site. The pooled results demonstrated significant differences between the remimazolam and propofol groups based on the incidence of hypotension, hypoxia, and pain of injection site; the outcomes in the remimazolam group were more favorable [hypotension: OR = 0.25, 95% CI (0.18, 0.34), *I*^2^ = 36%, *p* < 0.00001; hypoxia: OR = 0.15, 95% CI (0.07, 0.33), *I*^2^ = 0%, *p* < 0.00001; pain of injection site: OR = 0.03, 95% CI (0.01, 0.13), *I*^2^ = 0%, *p* < 0.0001, respectively]. There was no difference between the two groups based on the incidence of bradycardia, nausea, and vomiting. The pooled results demonstrated significant differences between the remimazolam and midazolam groups based on the incidence of hypotension; the remimazolam group had a better outcome [OR = 0.56, 95% CI (0.41, 0.77), *I*^2^ = 37%, *p* = 0.0003]. There was no difference between the two groups based on the incidence of hypoxia, bradycardia, nausea, vomiting, and pain at the injection site (Table 4).

**Table 4:** The pooled results of adverse events rates between remimazolam group and control group.

**Control**	**Complications**	**OR**	**95% CI**	***I*^**2**^**	***p*-value for effect**
Placebo	Hypotension	0.62	(0.42, 0.91)	36%	*p* = 0.01
	Hypoxia	1.03	(0.56, 1.87)	7%	*p* = 0.93
	Bradycardia	0.80	(0.41, 1.56)	0%	*p* = 0.51
	Nausea	0.51	(0.11, 2.46)	58%	*p* = 0.40
	Vomiting	0.59	(0.16, 2.21)	0%	*p* = 0.43
	Pain of injection site	0.99	(0.05, 20.82)	—	*p* = 0.99
Propofol	Hypotension	0.25	(0.18, 0.34)	36%	*p* <0.00001
	Hypoxia	0.15	(0.07, 0.33)	0%	*p* <0.00001
	Bradycardia	0.27	(0.06, 1.33)	—	*p* = 0.11
	Nausea	5.00	(0.58, 43.20)	—	*p* = 0.14
	Vomiting	2.95	(0.12, 72.95)	—	*p* = 0.51
	Pain of injection site	0.03	(0.01, 0.13)	0%	*p* <0.0001
Midazolam	Hypotension	0.56	(0.41, 0.77)	37%	*p* = 0.0003
	Hypoxia	1.04	(0.64, 1.68)	0%	*p* = 0.89
	Bradycardia	0.66	(0.38, 1.14)	0%	*p* = 0.14
	Nausea	1.13	(0.37, 3.43)	0%	*p* = 0.83
	Vomiting	1.01	(0.25, 4.07)	0%	*p* = 0.99
	Pain of injection site	1.15	(0.05, 24.28)	—	*p* = 0.93

—*: Represents only one study, the result has no I^2^ value. The adverse events was defined as follows: (1) hypotension: the reduction of SBP ≥ 20% or decreased to ≤ 80 mmHg (compared to baseline); (2) hypoxia: respiratory rate <8 breaths/min and/or oxygen saturation <90% in the duration from initial administration of trial drugs to fully alert; (3) bradycardia: heart rate <40 beats/min or a drop in heart rate of 20% or more from baseline, which lasted continuously for 30 s; (4) nausea, vomiting, and pain of injection site are considered to have occurred as long as the clinical symptoms appear more than once*.

## Discussion

This meta-analysis investigated the efficacy and safety of remimazolam sedation in endoscopy. Our results show that remimazolam had a strong sedative effect, and its sedative efficiency was significantly higher than that of placebo. Compared with the traditional sedative drugs, midazolam and propofol, the sedative efficiency of remimazolam was significantly higher than that of midazolam but lower than that of propofol. On the incidence of adverse events and complications, remimazolam was associated with a lower incidence of hypotension than placebo and midazolam, but there were no significant differences in hypoxia, bradycardia, nausea, vomiting, and pain at the injection site. Compared with propofol, remimazolam was associated with significantly lower incidence of hypotension, hypoxemia, and injection site pain but no differences in the incidence of bradycardia, nausea, and vomiting. Therefore, our results suggest that remimazolam has a good safety profile and a satisfactory efficacy for sedation for endoscopy.

Remimazolam, one of the newest benzodiazepines, acts on the gamma-aminobutyric acid receptor subunit (GABA_A_), and increasing the activity of the receptor exerts a sedative effect (25). It is an ultra-short-acting drug with a pharmacokinetic–pharmacodynamic profile characterized by rapid onset and recovery and moderate hemodynamic side effects (26). Remimazolam undergoes organ-independent metabolism and gets hydroxylated by plasma tissue esterases to an inactive metabolite, which allows for rapid removal even after use in prolonged infusions (24, 27). Therefore, prolonged infusions or high doses do not lead to drug or metabolite accumulation.

The clinical use of remimazolam has been considered in different settings. It has been evaluated as a premedication drug for use before anesthesia. However, its distinct bitter taste, very short duration of action, and low oral bioavailability limit its use in that regard (28). It has also been studied as a general anesthetic, using induction doses of 6 and 12 mg/kg/h and maintenance rates of 1 mg/kg/h. This demonstrated that remimazolam was non-inferior to propofol based on its efficacy as a general anesthetic, but the incidence of hypotension and other adverse events was significantly lower (29). Because of its ultra-short-acting and organ-independent metabolism characteristics, remimazolam has also been evaluated as a sedative agent for use in the ICU setting, making it an ideal agent for neurological evaluation soon after an infusion has been discontinued (30). However, no data are currently available for definitive conclusions.

Procedural sedation is widely used in endoscopic procedures around the world. Remimazolam has been studied for use in sedation for endoscopic procedures such as gastroscopy, colonoscopy, and bronchoscopy. Several original studies have shown that remimazolam facilitates faster onset and recovery, has higher sedative efficacy than midazolam, and is associated with lower incidence of hypotension and hypoxemia when compared with propofol (13, 14). This is consistent with the results of our meta-analysis, and this indicates that remimazolam has a better safety profile for sedation for endoscopic procedures.

In this article, we conducted a meta-analysis of different controls: placebo, midazolam, and propofol. On analyzing the sedative efficacy, we detected a high heterogeneity in the midazolam group, which could affect the reliability of the results of our meta-analysis. We conducted a sensitivity analysis to address the high heterogeneity using one-by-one literature exclusion (31). Consequently, three studies were excluded from the midazolam groups (that is, two studies each). We used a fixed-effect model to conduct meta-analyses for the remaining studies, and the pooled results were consistent with those before the sensitivity analysis. Another factor affecting the reliability of the results of the meta-analysis is publication bias (32). Since the number of included studies was low, we did not analyze publication bias in our meta-analysis; however, with the increase of related studies in the later period, further analysis is indispensable. Our meta-analysis included all relevant studies on remimazolam for use in sedation for endoscopic procedures; seven studies involving 1996 patients were included, and all of them were high-quality RCTs. We analyzed them separately according to the different control drugs, and the results were convincing and highly reliable.

However, our study still has the following limitations: (1) Remimazolam is a new drug, and the number of studies on its use in sedation for endoscopy is currently limited. With the increase in the number of studies, the sample size may have an impact on our results in the future. We will continue to pay attention to the research progress and update the results of the meta-analysis. (2) In the included studies, the doses of remimazolam were slightly different; two of the studies used a fixed dose, while the other five studies used intermittent additional doses based on the sedative effect. Therefore, our results cannot make valuable suggestions for the dose selection of remimazolam for endoscopic sedation. (3) The dose of the adjuvant opioid analgesics may not be consistent across the studies (fentanyl 0.5 μg/kg or a fixed dose of 50–100 μg), which may have affected our results. (4) The criteria for evaluating successful sedation in the seven studies were similar; however, two studies made appropriate adjustments, which may have affected our results. (5) The included studies are mainly concentrated in the United States and China, and the patient population may have limitations.

Given that remimazolam is an ultra-short-acting sedative, it has a good sedative efficiency and high safety for use in sedation for endoscopic procedures; its inhibitory effects on the respiratory and circulatory systems of the patients were significantly weaker than those of midazolam and propofol. Therefore, remimazolam may offer advantages in bronchoscopy sedation (more concerned about respiratory depression) over the currently used other. In addition, our results suggest that remimazolam may be safer for the sedation of older patients and those with poor cardiopulmonary function for endoscopic procedures.

## Conclusion

Remimazolam is a safe and effective sedative for patients undergoing endoscopic procedures. Its sedative efficiency was significantly higher than that of midazolam but slightly lower than that of propofol. However, its inhibitory effects on respiration and circulation are lesser than those of the aforementioned drugs. A few studies with small samples have reported the sedative efficiency of remimazolam for use in sedation for endoscopy and its associated incidence of adverse events, and the currently available data are insufficient to make conclusions. Therefore, high-quality RCTs with large samples are still needed in the future.

## Data Availability Statement

The original contributions presented in the study are included in the article/Supplementary Material, further inquiries can be directed to the corresponding author/s.

## Author Contributions

FY and ZW designed the meta-analysis and independently evaluated the methodological quality of included studies. HW and YJ performed the screening process for titles and abstracts, while XZ and SY performed the screening process for full texts. YL and ZZ supervised the acquisition and extracted data. HW conducted the statistical analysis of data. XZ wrote the manuscript. All authors contributed to the article and approved the submitted manuscript.

## Conflict of Interest

The authors declare that the research was conducted in the absence of any commercial or financial relationships that could be construed as a potential conflict of interest.

## Publisher's Note

All claims expressed in this article are solely those of the authors and do not necessarily represent those of their affiliated organizations, or those of the publisher, the editors and the reviewers. Any product that may be evaluated in this article, or claim that may be made by its manufacturer, is not guaranteed or endorsed by the publisher.
