# Supplementary material for: Efficacy and Safety of Remimazolam in Endoscopic Sedation—A Systematic Review and Meta-Analysis
Source: Front Med (Lausanne). 2021 Jul 26;8:655042. doi: 10.3389/fmed.2021.655042 (PMC8350069; doi:10.3389/fmed.2021.655042)
Supplement: Supplementary file 1 [file Table_1.DOC]

| **Section/topic** | **#** | **Checklist item** | **Reported on page #** |
| --- | --- | --- | --- |
| **TITLE** | | |  |
| Title | 1 | Efficacy and safety of remimazolam in endoscopic sedation - A systematic review and meta-analysis | P 1 |
| **ABSTRACT** | | |  |
| Structured summary | 2 | **Background:** The aim of this systematic review and meta-analysis was to investigate the efficacy and safety of remimazolam in clinical endoscopic procedures sedation.  **Methods:** Authors searched for studies that reporting remimazolam sedation in endoscopic procedures from the databases of Pubmed, Embase, and Cochrane Library until Jan 02, 2021. The sedative efficiency and the incidence of adverse events as outcome were assessed. Cochrane Review Manager Software 5.3 was used to perform statistical analyses.  **Results:** Seven relevant articles were identified and involving 1,996 patients. we conducted meta-analysis according to different control medications respectively, that is the placebo group, midazolam group and propofol group. The results demonstrated that remimazolam had a strong sedative effect, and its sedative efficiency is significantly higher compared to placebo (OR= 0.01, 95% CI: [0.00, 0.10], I2 =30%, p <0.00001). Compared with the traditional sedative drugs midazolam and propofol, the sedative efficiency of remimazolam was significantly stronger than midazolam (OR= 0.12, 95% CI: [0.08, 0.21], I2 =0%, p <0.00001), but weaker than propofol (OR= 12.22, 95% CI: [1.58, 94.47], I2 =0%, p =0.02). About adverse events, remimazolam has a lower incidence of hypotension compared to placebo and midazolam. Similarly, remimazolam had a lower incidence of hypotension and hypoxemia compared to propofol.  **Conclusions:** Remimazolam is a safe and effective sedative for the patients undergoing endoscopic procedures. The sedative efficiency of remimazolam was significantly stronger than midazolam, but slightly weaker than propofol. However, remimazolam's respiration and circulation inhibitory effect is smaller than midazolam and propofol. | P1-2 |
| **INTRODUCTION** | | |  |
| Rationale | 3 | Millions of patients worldwide receive endoscopy each year because of digestive tract or other discomforts. However, endoscopy is an invasive procedure after all, and patients may have many discomforts such as nervousness, fear, cough, gastrointestinal spasm, and even lead to serious complications such as arrhythmia and cerebrovascular accidents. Remimazolam, an analogue of midazolam, belongs to benzodiazepines and is a new ultra-short-acting sedative. Compared with midazolam, Remimazolam has the advantages of rapid onset, rapid recovery and higher safety. Previous studies have found that, in the endoscopic procedure sedation anesthesia, Remimazolam has the same success rate of sedation, lower incidence of hypotension and hyoxemia, and faster awakening time than propofol. However, as a new drug, the efficacy and safety of remimazolam for endoscopic sedation is still not completely clear. Therefore, we collected previously published relevant data to conduct a systematic review and meta-analysis on the efficacy and safety of remimazolam sedation in endoscopy. | P 2 |
| Objectives | 4 | The aim of this systematic review and meta-analysis was to investigate the efficacy and safety of remimazolam in clinical endoscopic procedures sedation. | P 2 and 3 |
| **METHODS** | | |  |
| Protocol and registration | 5 | No registration |  |
| Eligibility criteria | 6 | The inclusion criteria included (1) participants undergoing endoscopic procedures, including gastroscopy, colonoscopy, gastrointestinal endoscopy, and bronchoscopy. (2) sedation with remimazolam and placebo or other positive control agents. The exclusion criteria included (1) participants undergoing endoscopic procedures with unclear anesthetics; (2) duplicate articles (3) review or meta-analysis; (4) basic research; (5) article published as abstract, editorial, case report, letter, note, conference article, method or protocol; and (6) article presented in non-English language. | P 3 |
| Information sources | 7 | We searched the databases of Cochrane Library, Embase, Pubmed using the PICOS (Population, Intervention, Comparison, Outcome, Study design) method. The deadline for our search is January 02, 2021. | P 2-3 |
| Search | 8 | The search terms included “Remimazolam” OR “CNS 7056” AND “Endoscopy” OR “Bronchoscopy” OR “Colonoscopy” OR “Gastroscopy”, and the search scope was “title and abstract.” Because we sought to test all studies about the efficacy and safety of remimazolam for an endoscopy, we did not constrain the search terms of control drugs and study designs. Articles published in various languages will be included. Manual search will be performed on references lists of reviews and research papers to avoid any missing RCTs. | P 3 |
| Study selection | 9 | The inclusion criteria included (1) participants undergoing endoscopic procedures, including gastroscopy, colonoscopy, gastrointestinal endoscopy, and bronchoscopy. (2) sedation with remimazolam and placebo or other positive control agents. The exclusion criteria included (1) participants undergoing endoscopic procedures with unclear anesthetics; (2) duplicate articles (3) review or meta-analysis; (4) basic research; (5) article published as abstract, editorial, case report, letter, note, conference article, method or protocol; and (6) article presented in non-English language. | P 3 |
| Data collection process | 10 | Two authors were independently responsible for reviewing the titles, abstracts or both and summarized the data of the included literatures. Another two authors were performed the screening process for full texts | P 3 |
| Data items | 11 | (1) author; (2) publication year; (3) the number of participants in each study; (4) country of publication; (5) age range of all the participants; (6) gender composition; (7) the procedures that participants underwent; (8) the specific interventions that participants received, including the drug name, dose, and the medication regimen; (9) the methods and criteria for sedative efficacy assessment; (10) number of patients in remimazolam and control group. | P 3 |
| Risk of bias in individual studies | 12 | Two authors independently assessed the quality of included studies. Risk of bias was assessed using the Cochrane Collaboration Risk of Bias Assessment tool. They included the following seven items: random sequence generation (selection bias), allocation concealment (selection bias), blinding of participants and personnel (performance bias), blinding of outcome assessment (detection bias), incomplete outcome data (attrition bias), selective reporting (reporting bias), and others (bias due to vested financial interest and academic bias). If the study has one or more items associated with a high or unclear risk of bias, this trial was classified as high risk (16). If the two authors disagreed on their assessment, the corresponding author would be associated with other authors arbitrate any discrepancies to avoid the bias. | P 3-4 |
| Summary measures | 13 | The dichotomous outcome was reported as odds ratios (OR) with 95% confidence interval (CI). The statistical tests were two-sided and *p* value for overall effect <0.05 was considered significant differences. | P 4 |
| Synthesis of results | 14 | We used the values of I^2^ and the Mantel-Haenszel chi-square test (p-value for heterogeneity) to assess inter-study heterogeneity. The values of I^2^ < 40%, 40≤I^2^ < 60%, and I^2^ ≥60% indicated low, moderate, and high heterogeneity respectively. If significant heterogeneity was detected (I^2^≥50%), a leave-one-out sensitivity analysis was performed to assess the single comparison driven inference. Meta-analysis will be performed with random-effect model when there is insignificant heterogeneity (I^2^≥50% or a p-value for heterogeneity < 0.1), otherwise it will be performed with fixed-effect model (I^2^ < 50% or a p-value for heterogeneity≥0.1) | P 4 |

Page 1 of 2

| **Section/topic** | **#** | **Checklist item** | **Reported on page #** |
| --- | --- | --- | --- |
| Risk of bias across studies | 15 | Risk of bias was assessed using the Cochrane Collaboration Risk of Bias Assessment tool. If the study has one or more items associated with a high or unclear risk of bias, this trial was classified as high risk. If the two authors disagreed on their assessment, the corresponding author would be associated with other authors arbitrate any discrepancies to avoid the bias. | P3-4 |
| Additional analyses | 16 | The method of leave-one-out article sensitivity analysis was used to decrease the high heterogeneity. | P4 |
| **RESULTS** | | |  |
| Study selection | 17 | See Fig. 1 | P4 |
| Study characteristics | 18 | Seven articles were included with a total of 1996 patients and all were RCTs (published April 2005–Jan 2021). Four studies with 1079 patients undergoing colonoscopy, two studies with 478 patients undergoing upper gastrointestinal endoscopy, and one studies with 439 patients undergoing bronchoscopy. The age of the patients ranged from 18 to 95 years and male patients account for 45.38% (Table 1). Seven studies adopted the same or similar criteria to assess sedative efficiency. We assigned the patients in each study into two groups according to the type of sedative drugs in endoscopy: the remimazolam group and control groups (including placebo, midazolam and propofol). The proportion of patients with successful sedation was 1071/1208 in the remimazolam group and 481/788 in the control group (placebo 4/139, midazolam 88/270, and propofol 379/379, respectively) (Table 2). In addition, the incidence of adverse events, especially hypotension and hypoxia has been widely recorded (Table 3). | P 4 |
| Risk of bias within studies | 19 | The included seven studies demonstrated low risk of bias, as they clearly assessed the random sequence generation (seven studies, 100%), allocation concealment (seven studies, 100%), blinding of participants and personnel (six studies, 85.7%), blinding of outcome assessment (six studies, 85.7%), incomplete outcome data (seven studies, 100%), selective reporting (seven studies, 100%), and others (six studies, 85.7%). Among these studies, six studies were found to be high quality (Fig. 2 and Fig. 3). | P 4-5 |
| Results of individual studies | 20 | For all outcomes considered (benefits or harms), present, for each study: (a) simple summary data for each intervention group (b) effect estimates and confidence intervals, ideally with a forest plot. | P 5 |
| Synthesis of results | 21 | The fixed-effect model with OR was selected to assess the sedative efficacy of remimazolam and controlled drugs, and the pooled result demonstrated significant differences between remimazolam and placebo (OR= 0.01, 95% CI: [0.00, 0.10], I^2^ =30%, p <0.00001), propofol (OR= 12.22, 95% CI: [1.58, 94.47], I^2^ =0%, p =0.02), and, midazolam (OR= 0.12, 95% CI: [0.08, 0.21], I^2^ =0%, p <0.00001). | P 5 |
| Risk of bias across studies | 22 | Present results of any assessment of risk of bias across studies (see Item 15). | P 5 |
| Additional analysis | 23 | The method of leave-one-out article sensitivity analysis was used to decrease the high heterogeneity. Due to the noted significant heterogeneity between the included studies (I^2^=92%), leave-one-out analysis was performed. When the three studies (Keith 2015, Rex 2018 and Rex 2020) were excluded from the analysis, there was still significant difference between the two groups, and sedative efficacy was favoring the remimazolam group (OR= 0.12, 95% CI: [0.08, 0.21], I^2^ =0%, p <0.00001) (Fig. 7). | P 5 |
| **DISCUSSION** | | |  |
| Summary of evidence | 24 | Remimazolam, one of the newest benzodiazepines, acts on the gamma-aminobutyri cacid receptor subunit (GABAA) and increasing the activity of receptor exerts a sedative effect. It is an ultra-short acting drug with characterized by a pharmacokinetic–pharmacodynamic profile with fast onset, fast recovery, and moderate hemodynamic side effects. Remimazolam is organ-independent metabolism and to be hydroxylated by plasma tissue esterases to an inactive metabolite, which allows for rapid removal even is used in prolonged infusions. Therefore, prolonged infusions or high doses not lead to the drug or metabolites accumulation. Procedural sedation is widely used in the endoscopic procedures around the world. Remimazolam has been studied in endoscopic procedures sedation like gastroscopy, colonoscopy and bronchoscopy. Several original studies shown that remimazolam allows for a faster onset and recover time, stronger sedative efficacy when compared with midazolam, and a lower incidence of hypotension, hypoxemia when compared with propofol. This is consistent with our results of meta-analysis, and indicates that remimazolam has higher safety in procedural sedation for endoscopic procedures. | P 6 |
| Limitations | 25 | Since remimazolam is a new drug, the number of relevant studies is relatively small. As the number of remimazolam's studies on endoscopic procedures sedation increases in the future, it may have an impact on our outcome. In addition, the dose and type of adjuvant opioid analgesics between different studies may not be completely consistent, which may have a certain impact on our results. | P 7 |
| Conclusions | 26 | Remimazolam is a safe and effective sedative for the patients undergoing endoscopic procedures. Compared with the traditional sedative drugs, the sedative efficiency of remimazolam was significantly stronger than midazolam, but slightly weaker than propofol. However, remimazolam's respiration and circulation inhibitory effect is smaller than those of aforementioned drugs. Because a limited number of studies with a small sample size reported the sedative efficiency and incidence of adverse events for remimazolam sedation in endoscopic procedures, the current data were insufficient to make the conclusions. Therefore, high-quality RCTs with large samples are still needed in the future. | P 7 |
| **FUNDING** | | |  |
| Funding | 27 | This work was funded by the National Natural Science Foundation of China (Grant No. 81760257). | P 8 |

*From:*  Moher D, Liberati A, Tetzlaff J, Altman DG, The PRISMA Group (2009). Preferred Reporting Items for Systematic Reviews and Meta-Analyses: The PRISMA Statement. PLoS Med 6(7): e1000097. doi:10.1371/journal.pmed1000097

For more information, visit: **www.prisma-statement.org**.

Page 2 of 2
